# Exendin-4 regulates the MAPK and WNT signaling pathways to alleviate the osteogenic inhibition of periodontal ligament stem cells in a high glucose environment

**DOI:** 10.1515/med-2023-0692

**Published:** 2023-04-06

**Authors:** Min Wang, Min Liu, Jiawen Zheng, Li Xiong, Ping Wang

**Affiliations:** Department of Stomatology, The First Affiliated Hospital of Chongqing Medical University, Chongqing, 400016, China; Department of Pharmacy, The First Affiliated Hospital of Chongqing Medical University, Chongqing, China; Department of Stomatology, The First Affiliated Hospital of Chongqing Medical University, Youyi Road 1, Chongqing, 400016, China

**Keywords:** Exendin-4, osteogenic differentiation, PDLSCs, high glucose, MAPK signaling, WNT signaling

## Abstract

Diabetes mellitus (DM) increases the destruction of periodontal tissue and impairs osteogenesis differentiation. Exendin-4 (Ex-4), a glucagon-like peptide-1 (GLP-1) analogue, can be used for treating DM and promotes bone regeneration. The aim of this study was to explore the effect and mechanism of Ex-4 on improving the osteogenesis of periodontal ligament stem cells (PDLSCs) in a high glucose environment. Alkaline phosphatase staining and alizarin red staining were used to detect the osteogenic differentiation of PDLSCs. The results showed that 10 nM Ex-4 could reduce the osteogenesis inhibition of PDLSCs induced by high glucose. RT-PCR and western blot results showed that Ex-4 increased the osteogenesis-related gene expression of ALP, Runx2, and Osx, and upregulated the phosphorylation of P38, JNK, and ERK1/2; the peak effect was observed in the range 0.5–1.0 h. Mitogen-activated protein kinase (MAPK) inhibitors PD98059, SB203580, and SP600125 blocked the effects of Ex-4 on MAPK activation and decreased the expression of ALP, Runx2, and Osx in PDLSCs. Moreover, after Ex-4 treatment, the total β-catenin, p-GSK3β, LEF, and Runx2 protein levels increased under normal or high glucose environments. In conclusion, our results indicated that Ex-4 regulates the MAPK and WNT signaling pathways to alleviate the osteogenic inhibition of PDLSCs in a high glucose environment.

## 1 Introduction

Periodontitis is a destructive inflammatory disease caused by biofilm plaques; it destroys periodontal supporting tissues, including gums, periodontal membranes, alveolar bones, and cementum, and it is the main cause of adult tooth loss [1,2]. Currently, the main treatments for patients with periodontitis include periodontal scaling, flap turnover, and guided tissue regeneration. With the increasingly rapid progress of tissue engineering, periodontal regeneration has become a popular research area [3,4]. Periodontal ligament stem cells (PDLSCs) are the most promising seed cells in periodontal tissue engineering because of their easy sampling and multi-directional differentiation potential [5]. However, periodontitis is often accompanied by diabetes, and studies have shown that the prevalence of diabetes in patients with periodontitis is the highest in Asian countries (17.2%, *n* = 16,647) [6]. Diabetic status aggravates periodontal damage and hinders the repair of periodontal tissues, thus decreasing the regeneration rate in tissue engineering [7,8,9]. Therefore, determining how to enhance the osteogenic ability of PDLSCs in a high glucose environment is important for periodontal tissue repair and regeneration.

GLP-1 (glucagon-like peptide 1) is a kind of incretin synthesized and secreted by L cells of the intestine stimulated by food. It has a wide range of physiological functions, such as promoting the synthesis and release of insulin and reducing blood sugar concentration. However, GLP-1 is extremely unstable *in vivo*, easily degraded by DPP-4, and its serum half-life *in vivo* is only 2 min; therefore, its action time is very short [10]. Exendin-4 (Ex-4) is a peptide hormone, which was originally isolated from salivary secretions of Heloderma suspectum and has 53% homology with GLP-1. It stimulates the secretion of insulin by pancreatic β-cells, thereby controlling blood glucose levels. *In vivo*, Ex-4 has a similar biological function to that of GLP-1 but its half-life is longer and its degradation tendency is lower, so it is currently widely used in the clinical treatment of type 2 diabetes [11]. Recent reports have demonstrated that Ex-4 is an important regulator of bone growth and remodeling. Animal experiments have shown that Ex-4 and GLP-1 receptors promote the osteogenic differentiation of bone marrow stem cells and MC3T3-E1 preosteoblasts [12,13,14]. A recent study has also demonstrated that 10 nmol/L Ex-4 promotes migration and osteogenic differentiation of PDLSCs [15]. Our preliminary experiments have shown that the osteogenic inhibition induced by high glucose in PDLSCs is alleviated by 10 nM Ex-4 [16], although the molecular pathways involved in PDLSCs remain unclear. mitogen-activated protein kinase (MAPK) and WNT pathways play an important role in many biological processes such as cell growth, development, and metabolic balance. It is found that the MAPK pathway is particularly important for the differentiation of osteoblasts and the regulation and control of bone development [17]. Ex-4 can promote the osteogenic differentiation of MC3T3-E1 cells by activating the MAPK pathway [12]. The Wnt/β-catenin pathway can promote the differentiation of osteoblasts by upregulating osteogenic regulatory factors and also plays an important role in regulating bone development and increasing bone mass [18]. Therefore, we hypothesized that Ex-4 may promote the osteogenesis of PDLSCs in a high glucose environment by regulating MAPK and WNT pathways.

The purpose of this study was to explore the osteogenesis regulatory mechanisms of Ex-4 in PDLSCs under high glucose conditions and to provide a new method for the clinical treatment of diabetic periodontitis.

## 2 Materials and methods

### 2.1 Isolation and culture of PDLSCs

The premolars of healthy people were collected from orthodontic patients (18–26 years of age) who visited the Department of Orthodontics of the First Affiliated Hospital of Chongqing Medical University. Consent was obtained from all patients. After the removal of excess epithelial and granulation tissue, the teeth were washed repeatedly with phosphate-buffered saline (PBS; Hyclone, USA). The periodontal membrane was scraped softly from a third of the root and then digested with collagenase type I (3 mg/mL, Sigma-Aldrich, USA) in a water bath at 37°C for 10 min. Finally, the tissues were transferred to 25 T cell culture flasks containing fetal bovine serum (Hyclone, USA) at 37°C in a humidified atmosphere containing 5% CO_2_. After culturing for 4 h, alpha modification of eagle’s medium (α-MEM) (Gibco, USA) supplemented with 10% fetal bovine serum (FBS) and 1% streptomycin solution (Sigma-Aldrich, USA) was added to the culture medium and changed every 3 days. PDLSCs from P3 to P5 were used for subsequent experiments.


**Ethics approval and consent to participate:** This study was carried out in accordance with the Declaration of Helsinki principles and the guidelines of the Ethics Committee of the First Affiliated Hospital of Chongqing Medical University, and the approval reference number is 201810401. Furthermore, all subjects have given their written informed consent.

### 2.2 Identification of PDLSCs

To determine the characteristics of PDLSCs, we detected the colony-forming ability and multi-directional differentiation ability of PDLSCs. PDLSCs were inoculated into 6 cm^2^ cell culture dishes. They were then cultured in a growth medium for 14 days, and about 2 mL of crystal violet dye solution (Beyotime, China) was added to each dish and the clone formation rate was calculated. When the number of cells in an aggregation exceeded 50, it was scored as a colony. For osteogenic experiments, PDLSCs were cultured in an osteogenic induction medium comprising α-MEM, 10% FBS, 1% streptomycin solution, 1 µM dexamethasone, 50 µg/mL ascorbic acid, and 3 µM β-glycerophosphate (Sigma-Aldrich, USA). After 21 days, alizarin red staining was used to assess the degree of osteogenic differentiation. For adipogenesis, PDLSCs were cultured in an adipogenic induction medium consisting of α-MEM, 10% FBS, 1% streptomycin solution, 1 µM dexamethasone, 10 µM insulin, 200 µM indomethacin, and 0.5 mM 3-isobutyl-1-methylxanthine (all from Sigma-Aldrich, USA). After 14 days, Oil Red O staining was used to detect the lipid droplets.

### 2.3 Alkaline phosphatase staining

PDLSCs were induced in an osteogenic induction medium containing 5.5 mM d-glucose (Sigma, USA) (NG), NG + 10 nM Ex-4 (NG + Ex-4), 30 mM d-glucose (HG), or HG + 10 nM Ex-4 (HG + Ex-4) for 7 days. ALP staining solution (Beyotime, China) was prepared according to the protocol of the alkaline phosphatase staining kit, and 500 μL of the ALP staining solution was added to the well to be tested, and incubated for 15 min at room temperature in the dark. Subsequently, all staining solution was aspirated, and the well was gently rinsed with ultrapure water, photographed, and observed.

### 2.4 Alizarin red staining

PDLSCs were cultured in the osteogenic induction medium for 21 days. Then, the cells were fixed with 4% paraformaldehyde at room temperature in the dark for 30 min and stained with alizarin red (Suolaibao, Beijing, China) for 30 min; the dye solution was then discarded. After careful washing with PBS three times, the cells were observed and photographed under an inverted microscope.

### 2.5 Quantitative real-time polymerase chain reaction (qRT-PCR)

PDLSCs were osteogenically cultured with NG or 30 mM mannitol for 7 days. PDLSCs were also cultured with NG + 10 nM Ex-4, HG, or HG + 10 nM Ex-4 for 7 or 14 days, and osteogenesis was induced. The MAPK-specific inhibitors, 25 µM PD98059, 25 µM SB203580, and 10 µM SP600125 (MCE, USA), were also used to treat the cells. The expression of ALP, Osx, and Runx2 was detected with RT-PCR. Total RNA was extracted from the PDLSCs with TRIzol reagent (Invitrogen, USA). RNA (1,000 ng) was then transcribed into cDNA using a PrimeScript™ RT reagent kit with gDNA Eraser (Takara, Japan). Finally, an SYBR Premix Ex Taq II kit (Takara, Japan) was used for RT-PCR. A total of 10 µL PCR system contained 2 µL of cDNA, 0.5 µL of each diluted primer, 5 µL of SYBR green, and 2 µL of distilled water. The primer sequences used in this research are listed in Table 1. The results were analyzed with the comparative 2^−ΔΔCt^ method.

**Table 1:** Sense and antisense primer sequences used for real-time PCR

Primers	Sequences
Runx2	Forward (5′–3′): GTCTCACTGCCTCTCACTTG
	Reverse (5′–3′): CACACATCTCCTCCCTTCTG
ALP	Forward (5′–3′): CCATACAGGATGGCAGTGAAGG
	Reverse (5′–3′): TTGACCTCCTCGGAAGACACTC
Osx	Forward (5′–3′): TGAGGAGGAAGTTCACTATGG
	Reverse (5′–3′): TTCTTTGTGCCTGCTTTGC
β-Actin	Forward (5′–3′): CCTGGCACCCAGCACAAT
	Reverse (5′–3′): GGGCCGGACTCGTCATAC

### 2.6 Western blot analysis

The levels of key proteins in MAPK and WNT pathways were tested by western blotting. PDLSCs were treated with 10 nM Ex-4 for 0, 0.5, 1, 1.5, 2, or 3 h. For inhibition experiments, cells were pretreated with the specific inhibitors PD98059 (25 µM), SB203580 (25 µM), or SP600125 (10 µM) (MCE, USA) for 4 h, then incubated with Ex-4 (10 nM) for 0.5 or 1 h, and cellular proteins were extracted. A BCA protein assay kit (Beyotime Biotechnology) was used to detect and quantify the protein concentrations of PDLSCs. Then, 20 μg of total protein was separated with 12% SDS-PAGE and transferred to polyvinylidene fluoride membranes (Millipore, USA). The membrane was blocked in 5% skim milk and incubated on a shaker at room temperature for 2 h. Membranes were incubated overnight at 4°C with primary antibodies to p38, p-p38, ERK1/2, p-ERK1/2, JNK, p-JNK, GSK3β, p-GSK3β (Ser9), LEF, β-catenin, GAPDH, tubulin (Cell Signaling Technology, USA) and Runx2 (HuaBio, China) diluted 1:1,000, and was then incubated with secondary antibodies (Cell Signaling Technology, USA) for 1 h at room temperature. Proteins were visualized with Hypersensitive ECL Chemiluminescence kits (Beyotime, China) and analyzed using Image J software.

### 2.7 Statistical analyses

All statistical calculations were analyzed using Prism software (version 8.0). The differences between the two groups of data were compared with Student’s *t*-test, and multiple groups of data were evaluated with one-way ANOVA. Differences were considered significant at *p* < 0.05.

## 3 Results

### 3.1 Isolation and identification of PDLSCs

**Figure 1:**
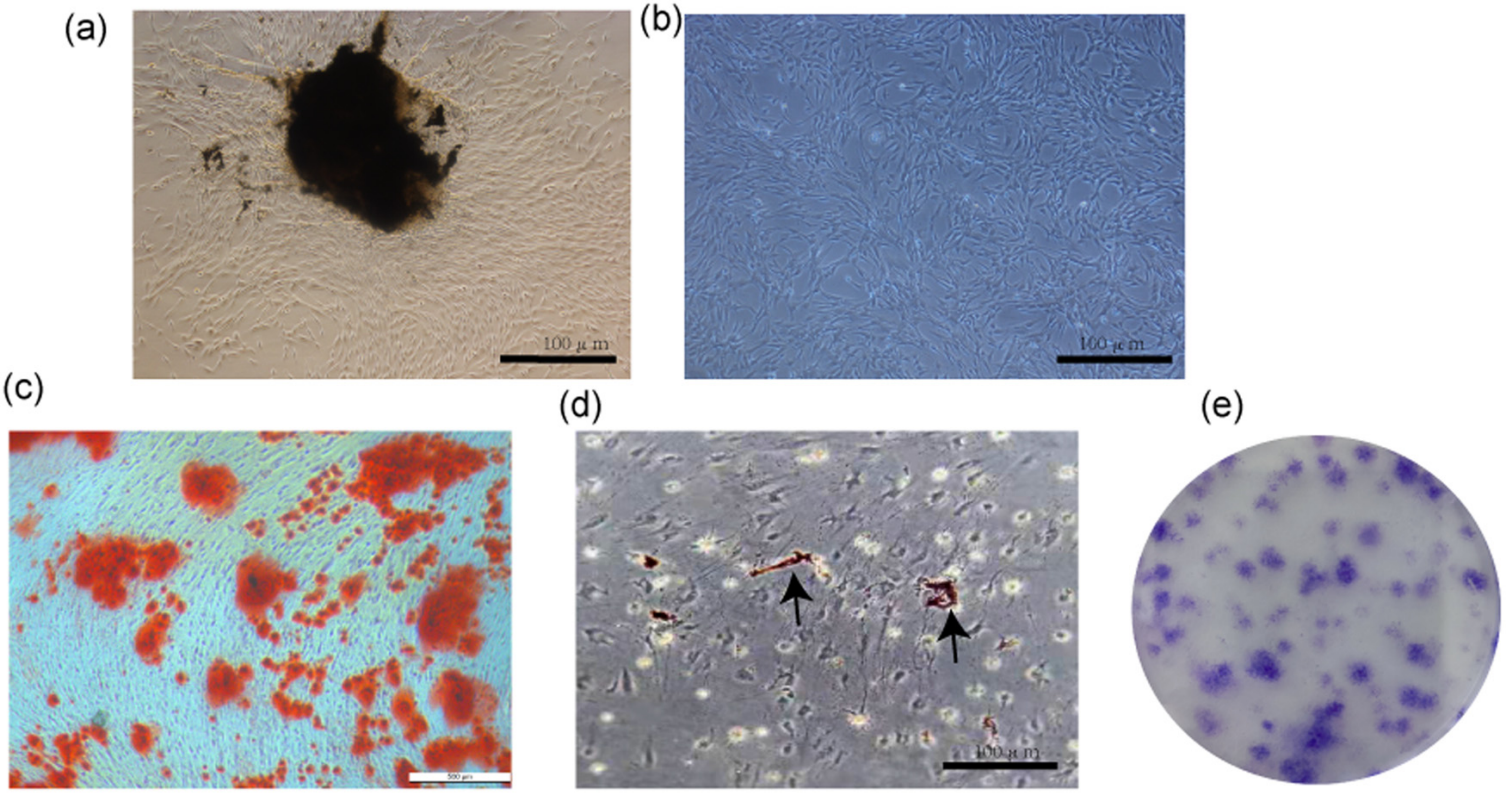
Extraction and identification of PDLSCs. (a) Primitive cells grow out of the surrounding tissue mass. (b) Morphology of PDLSCs. (c) PDLSCs formed calcium nodules stained with Alizarin Red S after 21 days of osteogenesis induction. (d) After 14 days of induction of lipogenesis, lipid droplets were assessed by oil red o staining. (e) Plate clone formation experiment and crystal violet staining were carried out after 14 days of culture.

Primary periodontal ligament cells were successfully extracted from periodontal ligament tissue (Figure 1a). Under microscopic examination, the cells were seen to have grown in long spindle shapes (Figure 1b). At 21 days after osteogenesis induction, alizarin red staining showed that calcium nodules had formed (Figure 1c); 14 days after induction of adipogenesis, oil red O staining showed the formation of lipid droplets (Figure 1d). The PDLSCs formed high-density clones after 2 weeks of growth (Figure 1e). Our results indicated that PDLSCs had multi-directional differentiation potential.

### 3.2 Influence of Ex-4 on the osteogenic differentiation of PDLSCs under high glucose conditions

**Figure 2:**
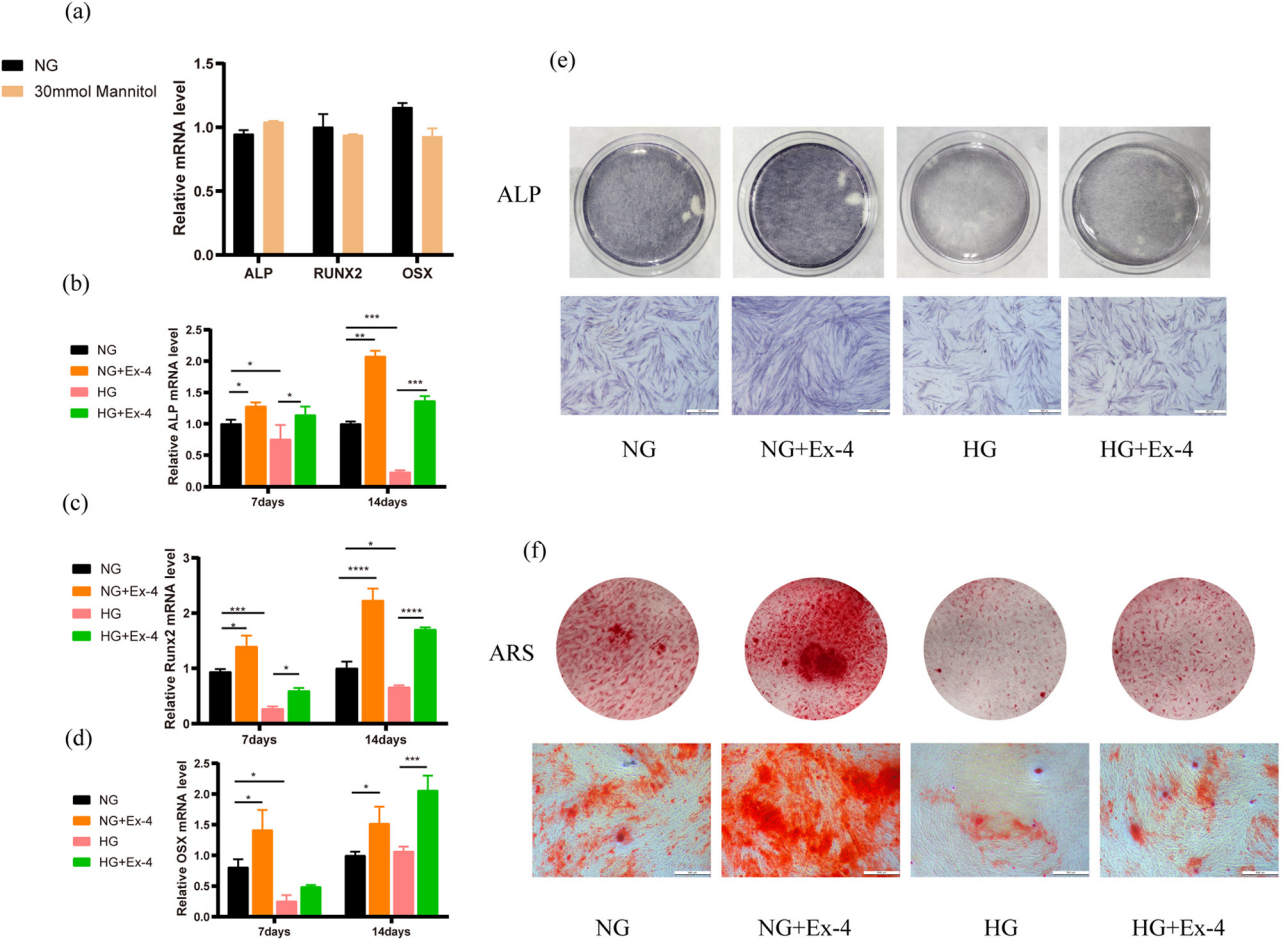
Influences of Ex-4 on the osteogenic differentiation of PDLSCs in a high glucose environment. (a) RT-PCR was used to detect the expression of ALP, Runx2, and Osx in PDLSCs cultured in 30 mmol mannitol for 7 days. (b–d) PDLSCs were osteogenically cultured for 7 and 14 days, and the expression of ALP, Osx, and Runx2 was detected with RT-PCR. (e) Alkaline phosphatase staining was used to detect the ALP activity of PDLSCs on the 7th day. Scale bars = 500 μm. (f) Mineralized nodules were detected with alizarin red staining on the 21st day. Nodules (15×) were observed under a microscope. Scale bars = 500 μm. Data are expressed as the average of three independent experiments + SEM (*p < 0.05, **p < 0.01, ***p < 0.001, and ****p < 0.0001).

Ex-4 is used as an antidiabetic agent in clinical settings, and studies have shown that Ex-4 promotes osteogenesis. Therefore, we investigated whether Ex-4 promotes the osteogenesis of PDLSCs under high glucose conditions. In order to eliminate the influence of osmotic pressure on the osteogenic ability of PDLSCs, PDLSCs were cultured in an osteoblast induction medium and treated with mannitol (30 mM) for 7 days. RT-PCR indicated no significant differences in the expression of osteogenic differentiation-associated genes (including Runx2, ALP, and Osx) between the 30 mM mannitol group and control group (*p* > 0.05) (Figure 2a). Our findings demonstrated that the inhibition of PDLSC osteogenesis by high glucose was independent of osmotic pressure.

We then used 10 nM Ex-4 to treat PDLSCs in normal or high glucose environments. After 7 and 14 days of osteogenic culture, we detected the changes in osteogenesis-related gene expression by RT-PCR. The results showed that mRNA expression of osteogenic genes (ALP, Runx2, and Osx) after treatment with 10 nM Ex-4 was markedly higher than that of the control group on the 14th day (*p* < 0.01). Under HG conditions, the mRNA levels of ALP, Osx, and Runx2 were lower than that of the control group on the 7th day, and the mRNA levels of ALP and Runx2 were markedly lower than that of the control group on the 14th day (*p* < 0.05). The RT-PCR results also indicated that the effects of inhibition of high glucose on osteogenic genes in PDLSCs were clearly decreased by co-treatment with Ex-4 for 14 days (*p* < 0.01) (Figure 2b–d). These results further indicated that Ex-4 has a positive effect on the osteogenesis of PDLSCs in a high glucose environment.

We further detected the changes in osteogenesis by ALP staining and alizarin red staining. After 7 and 21 days of osteogenic culture, the results indicated that the ALP activity and number of calcium nodules formed in the high glucose group strongly decreased. After treatment with Ex-4, the ALP activity and the number of calcium nodules in HG + Ex-4 were greater than those in the HG group (Figure 2e and f). These results indicated that Ex-4 alleviated the osteogenic inhibition of PDLSCs under a high glucose environment.

### 3.3 Influence of Ex-4 on MAPK signaling in PDLSCs

**Figure 3:**
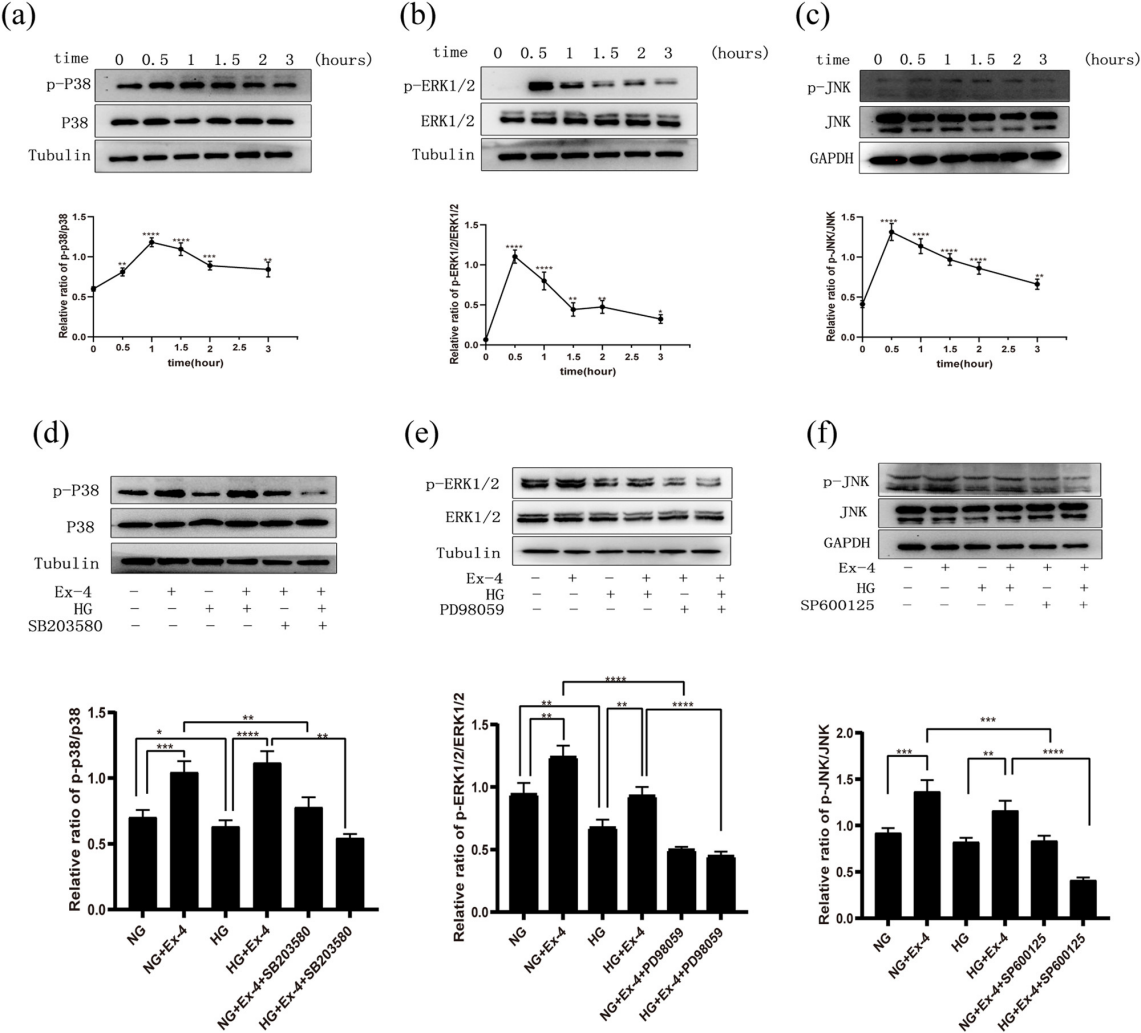
Influences of Ex-4 on MAPK phosphorylation in PDLSCs. (a–c) The phosphorylation of p38, ERK1/2, and JNK was detected by WB after 0, 0.5, 1, 1.5, 2, and 3 h of Ex-4 treatment of PDLSCs, compared with 0 h. (d–f) Cells were pretreated with 25 μM PD98059, 25 μM SB203580, or 10 μM SP600125 before incubation with 10 nM Ex-4 or 30 mM HG for 0.5 h. Total proteins were extracted to detect the phosphorylation of p38, ERK1/2, and JNK. Data are expressed as the average of three independent experiments + SEM (*p < 0.05, **p < 0.01, ***p < 0.001, and ****p < 0.0001).

GLP-1 and its analogues have been shown to affect osteoblasts through MAPK pathways. Therefore, we explored the possible role of the MAPK pathway in Ex-4’s regulation of the osteogenic differentiation of PDLSCs. After Ex-4 treatment, the expression of three MAPK kinases (p38, ERK1/2, and p38) and their phosphorylation were detected at different times. Western blotting indicated that Ex-4 promoted the phosphorylation of MAPKs in PDLSCs between 0.5 and 1.0 h. The phosphorylation of p38 peaked around 1.0 h, those of extracellular signal-regulated kinase (ERK) and c-Jun N-terminal kinase (JNK) both peaked around 0.5 h (*p* < 0.01) (Figure 3a–c).

To further verify the effects of Ex-4 via the MAPK pathway, we pretreated cells with MAPK inhibitors (including 25 µM PD98059, 25 µM SP 600125, and 10 µM SB203580) for 4 h, and then treated the cells with 10 nM Ex-4 or 30 mM HG for approximately 30 min. Western blotting detection showed that Ex-4 promoted greater MAPK phosphorylation of ERK1/2, p38, and JNK than that in the control group, and the phosphorylation of p38 and ERK1/2 in the HG group decreased. Compared with that in the HG group, the phosphorylation of MAPKs in the HG + Ex-4 group was also elevated. Furthermore, MAPK inhibitor treatment significantly decreased the phosphorylation of MAPKs by Ex-4. Thus, Ex-4 activates the MAPK pathway in a high glucose environment (Figure 3d–f).

### 3.4 Influence of Ex-4 on osteogenesis in PDLSCs is blocked by MAPK inhibitors

**Figure 4:**
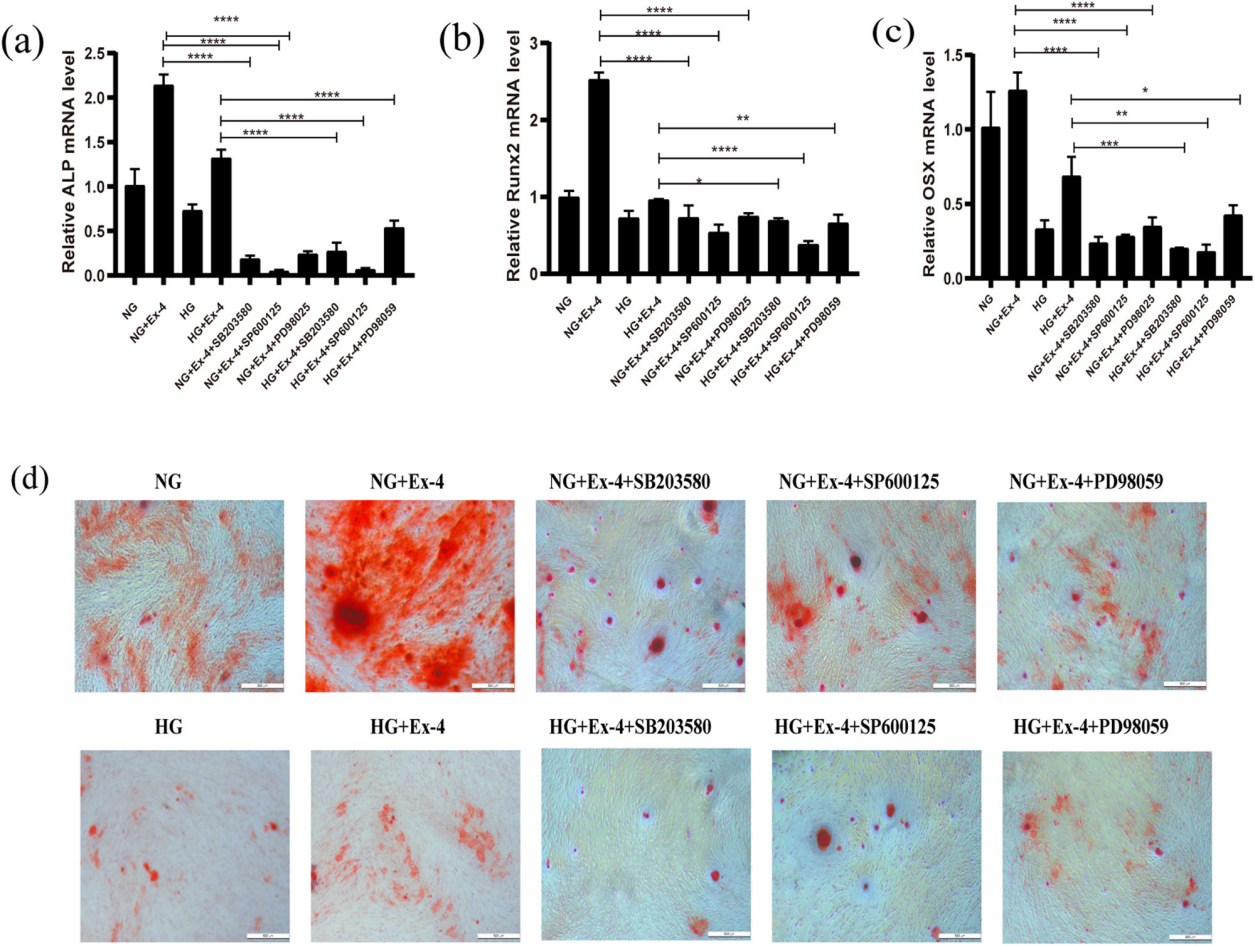
The influence of Ex-4 on osteogenesis in PDLSCs is blocked by MAPK inhibitors. (a–c) PDLSCs were switched to a differentiation medium and treated with PD98058, SB203580, or SP600125 in the presence or absence of 10 nM Ex-4 or 30 mM high glucose. Total RNA was extracted on day 7, and quantitative PCR was performed. (d) Mineralized nodules were detected with alizarin red staining, scale bars = 500 μm. Data are expressed as the average of three independent experiments + SEM (*p < 0.05, **p < 0.01, ***p < 0.001, ****p < 0.0001).

MAPK inhibitors were added, and their influence on the mRNA expression of ALP, Runx2, and Osx was assessed. Compared with those in the NG + Ex-4 group, the mRNA levels of ALP, Runx2, and Osx in the NG + Ex-4 + SB203580 group were 92, 72, and 82% lower, respectively. Compared with those in the HG + Ex-4 group, the levels were approximately 80, 69, and 71% lower in the HG + Ex-4 + SB203580, respectively (*p* < 0.01). Similarly, for SP600125, compared with the NG + Ex-4 group, the mRNA levels of ALP, Runx2, and OSX were reduced to approximately 98, 53, and 28%, respectively, in the NG + Ex-4 + SP600125 group, and compared with HG + Ex-4, they were reduced to about 95, 38, and 20% (*p* < 0.05) in the HG + Ex-4 + SP600125 group. PD98059 likewise reduced all the mRNA expressions; compared with the NG + Ex-4 group, it decreased the expressions of ALP, Runx2, and OSX to about 89, 70, and 73% in the NG + Ex-4 + PD98059 group, and compared with the HG + Ex-4 group the expressions decreased to 59, 65, and 38% in the HG + Ex-4 + PD98059 group, respectively (*p* < 0.05) (Figure 4a–c). Alizarin red staining indicated that the calcified nodules in the inhibitor groups were much lower than those in the control groups, thus further indicating that the MAPK inhibitor blocked the effect of Ex-4 on the osteogenesis of PDLSCs (Figure 4d).

### 3.5 Influence of Ex-4 on WNT signaling in PDLSCs under high glucose conditions

**Figure 5:**
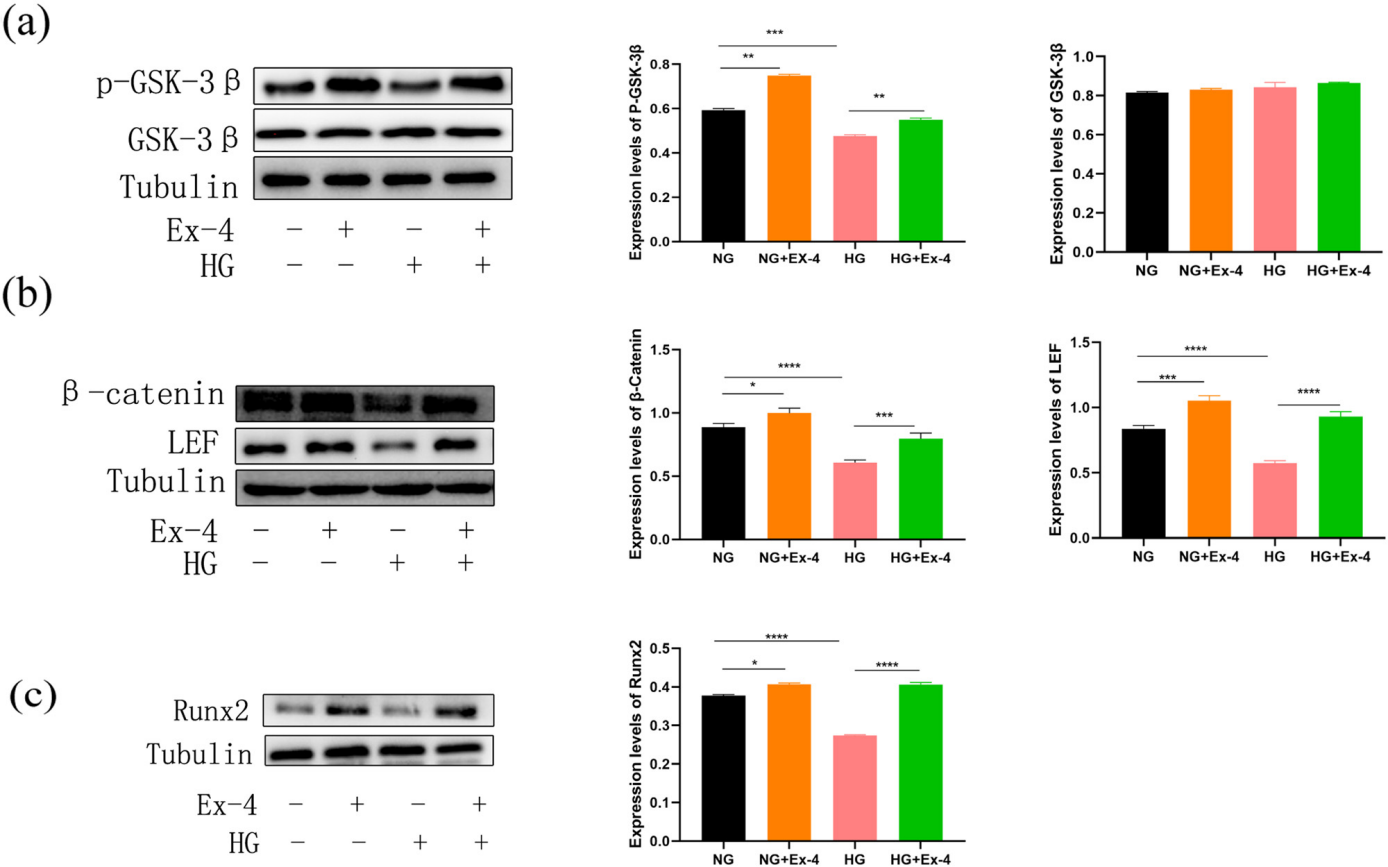
The influence of Ex-4 on WNT signaling in PDLSCs under high glucose conditions. (a–c) Western blots showing the expression of p-GSK3β, GSK3β, LEF, β-catenin, and Runx2 after PDLSC treatment with 10 nM Ex-4 or 30 mM HG in an osteogenic medium for 7 days. Data are expressed as the average of three independent experiments + SEM (*p < 0.05, **p < 0.01, ***p < 0.001, and ****p < 0.0001).

The WNT signaling pathway also plays an important role in the osteogenesis of stem cells, and studies have found that Ex-4 can promote the bone formation of type 2 diabetic rats through the WNT signaling pathway [19]. Therefore, we speculate that Ex-4 may promote the bone formation of PDLSCs in a high glucose environment through the WNT signaling pathway. PDLSCs were cultured in 30 mM high glucose or 10 nM Ex-4 for 7 days, and the protein levels of β-catenin, p-GSK-3β, GSK-3β, LEF, and Runx2 were detected. Western blotting showed that compared with the NG control group, p-GSK-3β was significantly higher in the NG + Ex-4 group and lower in the HG group. Compared with that in the HG group, the expression of p-GSK-3β in the HG + Ex-4 group was also higher, but the total protein of GSK-3β showed no clear difference (Figure 5a). The expression of total β-catenin and LEF increased after Ex-4 treatment, whereas that in the HG group decreased. Co-treatment with Ex-4 under high glucose conditions significantly increased β-catenin and LEF expression (Figure 5b). The expression of Runx2 in the NG + Ex-4 group was significantly higher, whereas that in the HG group was significantly lower than that in the NG control group. Co-treatment with Ex-4 under high glucose, compared with HG treatment alone, significantly increased Runx2 expression (*p* < 0.05) (Figure 5c).

## 4 Discussion

In the present study, we found the GLP1 receptor agonist Ex-4 exerted a direct action on PDLSCs in a high glucose environment. Ex-4 alleviated the osteogenic inhibition of PDLSCs induced by high glucose, and promoted the osteogenic differentiation of PDLSCs in a normal glucose environment. Ex-4 also upregulated the activity of MAPK and WNT signaling pathways, which are known to play a key role in osteoblast differentiation and bone development. Taken together, these results imply that Ex-4 may play an active role in the osteogenesis of PDLSCs by regulating MAPK and WNT pathways in a high glucose environment.

Studies have shown a bidirectional relationship between diabetes and periodontitis. For patients with periodontitis and diabetes, hyperglycemia can accelerate the destruction of periodontal tissue and promote alveolar bone absorption [20]. ALP is an enzyme secreted by osteoblasts, and its high activity and expression are the specific sign of early differentiation of osteoblasts [21]. Runx2 is one of the earliest and most specific marker genes involved in bone formation [22], and Osx is an osteoblast-specific transcription factor necessary for bone formation [23]. In the present study, we found that the expression of the osteogenic genes ALP, Runx2, and Osx in PDLSCs decreased under high glucose conditions and the calcium nodules also decreased, as detected by alizarin red staining. Therefore, our results showed that a high glucose environment damaged the osteogenesis of PDLSCs.

Ex-4, an analogue of GLP-1, is used as a drug to treat type 2 diabetes. Recent studies have demonstrated that GLP-1 and its analogues play active roles in bone metabolism and have attracted increasing attention. In animal experiments, Nuche-Berenguer et al. have found that GLP-1 and Ex-4 had osteogenic roles in diabetes and can reverse the osteopenia associated with hyperlipidemia [24]. Moreover, Ex-4 stimulates the secretion of insulin, inhibits the secretion of glucagon and regulates gastric emptying, controls glucose levels, and promotes bone formation [25,26]. In addition to indirectly inhibiting bone resorption by lowering blood sugar, Ex-4 also directly inhibits bone resorption and promotes bone formation, thereby increasing bone mass [27,28]. Here, *in vitro*, our data showed that 10 nM Ex-4 rapidly increased the expression of the osteogenic genes ALP, Runx2, and Osx. Ex-4 restored the expression of damaged osteogenic genes under high glucose conditions. Nonetheless, we conclude that Ex-4 has a positive effect on the PDLSC differentiation under a high glucose environment. This conclusion is in line with many previous research findings.

To further investigate the osteogenic mechanism of Ex-4 in PDLSCs, we studied the effects of Ex-4 on the activation of the MAPK and WNT signaling pathways. MAPK signaling pathways regulate a series of biological activities, such as growth, reproduction, and death. It is particularly important for the differentiation of osteoblasts and the regulation and control of bone development [17]. Four MAPK signal transduction pathways are known in eukaryotic cells: the ERK, JNK (also known as SAPK), and p38 and ERK5 pathways [29,30,31,32]. Recent studies have shown that GLP-1 and its analogues affect osteoblasts through MAPK pathways [12,33]. Feng et al. have confirmed that Ex-4 rapidly activated the MAPK pathway in MC3T3-E1 cells, whereas blocking the MAPK pathways with an inhibitor consequently inhibited osteogenesis [12]. According to our results, Ex-4 activated MAPK pathways in a short time, and the phosphorylation of ERK1/2 and JNK reached a peak at 0.5 h, and that of p38 reached its peak at 1 h. However, studies about the role of Ex-4 in the MAPK pathway under a high glucose environment have remained controversial. Several research studies have reported that Ex-4 downregulates the phosphorylation of ERK and p38 MAPK in peripheral blood mononuclear cells of patients with type 2 diabetes, so as to reduce oxidative stress and the pro-inflammatory reaction [34,35]. Conversely, results from other studies have demonstrated that Ex-4 can accelerate glucose transport in adipocytes by activating MAPKs [36]. Our data showed that phosphorylation of p38 and ERK1/2 decreased under a high glucose environment, but Ex-4 promoted the phosphorylation of the MAPK pathway in both normal and high glucose groups. To further confirm the role of MAPK pathway in the osteogenesis of PDLSCs, we chose an MAPK pathway inhibitor to act on PDLSCs. SB203580 is a p38-specific inhibitor, which can permeate cells and inhibit the subsequent activation of MAPKAPK-2 and MAP-KAPP-3, thus inhibiting the p38 pathway [37]. PD98059 is a selective inhibitor of ERK1/2; it binds to the upstream MEK1 protein kinase of ERK, thereby inhibiting its phosphorylation and blocking its signal transduction, thus inhibiting the activation of the downstream ERK1/2 [38]. SP600125 is a specific inhibitor of the JNK pathway; it reversibly competes with ATP, thus inhibiting the phosphorylation of JNK [39]. When we added ERK, JNK, and p38 blockers (PD98059, SP600125, and SB203580) under a high glucose environment, the ALP, Runx2, and Osx mRNA levels in PDLSCs significantly decreased. We suspected that ERK1/2 and P38 pathways of the MAPK pathway play a major role in the early osteogenesis of PDLSCs, while the JNK pathway may be mainly related to the inflammatory reaction in cells, or participate in the late osteogenic differentiation of PDLSCs. In conclusion, our results demonstrated that Ex-4 may promote the osteogenesis of PDLSCs by activating MAPK pathways under high glucose conditions. Furthermore, p38, ERK1/2, and JNK proteins are sensitive to intracellular oxidative stress [40,41]. People with diabetes have a complex internal environment. Advanced glycation end products formed by long-term hyperglycemia activate RAGE-mediated oxidative stress and then induce osteoblasts to produce ROS, thus inhibiting their proliferation and osteogenic differentiation [42]. Our research group has also found that Ex-4 regulates ROS levels in cells, thereby affecting PDLSC proliferation and osteogenesis [16]. Whether Ex-4 might regulate the MAPK pathway by decreasing ROS production to alleviate the osteogenesis inhibition of PDLSCs under high glucose requires further investigation.

The classical WNT signaling pathway is closely associated with the regulation of osteoblast differentiation. The pathway is mainly composed of ligands (WNT family molecules), transmembrane receptors (Frizzled family molecules and LRP-5/6), cytoplasmic regulatory proteins (Dsh, APC, Axin, GSK-3β, and β-catenin), and nuclear transcription factor families (TCF/LEF) [43]. GSK-3β, a very important negative regulator in the classic Wnt pathway, phosphorylates β-catenin, thus leading to the ubiquitination and degradation of β-catenin [44]. Runx2, a key gene in osteogenesis, plays a significant role in promoting osteogenesis through the classic WNT pathway [45,46]. Previous studies have found that Ex-4 treatment activates the WNT pathway to promote bone formation in diabetic rats. In addition, Kim et al. have further suggested that Ex-4 may bind the GLP-1 receptor in a manner mediated by protein kinase A and act on the WNT/β-catenin pathway in bone cells, thereby promoting bone formation [19]. Our results showed that after Ex-4 treatment, the levels of p-GSK3β, total β-catenin, and LEF increased under normal and high glucose environments. Thus, we concluded that Ex-4 renders the GSK3β-mediated complex ineffective by activating GSK3β phosphorylation, promoting β-catenin nuclear translocation and interaction with LEF, and finally, increasing the expression of the osteogenesis-related gene Runx2. However, our experiments have several limitations, we only selected a part of the WNT pathway proteins to study, but the expression changes in the β-catenin phosphorylation level were not further explored, and the relationship between Ex-4 and classical WNT pathway requires further research.

## 5 Conclusion

**Figure 6:**
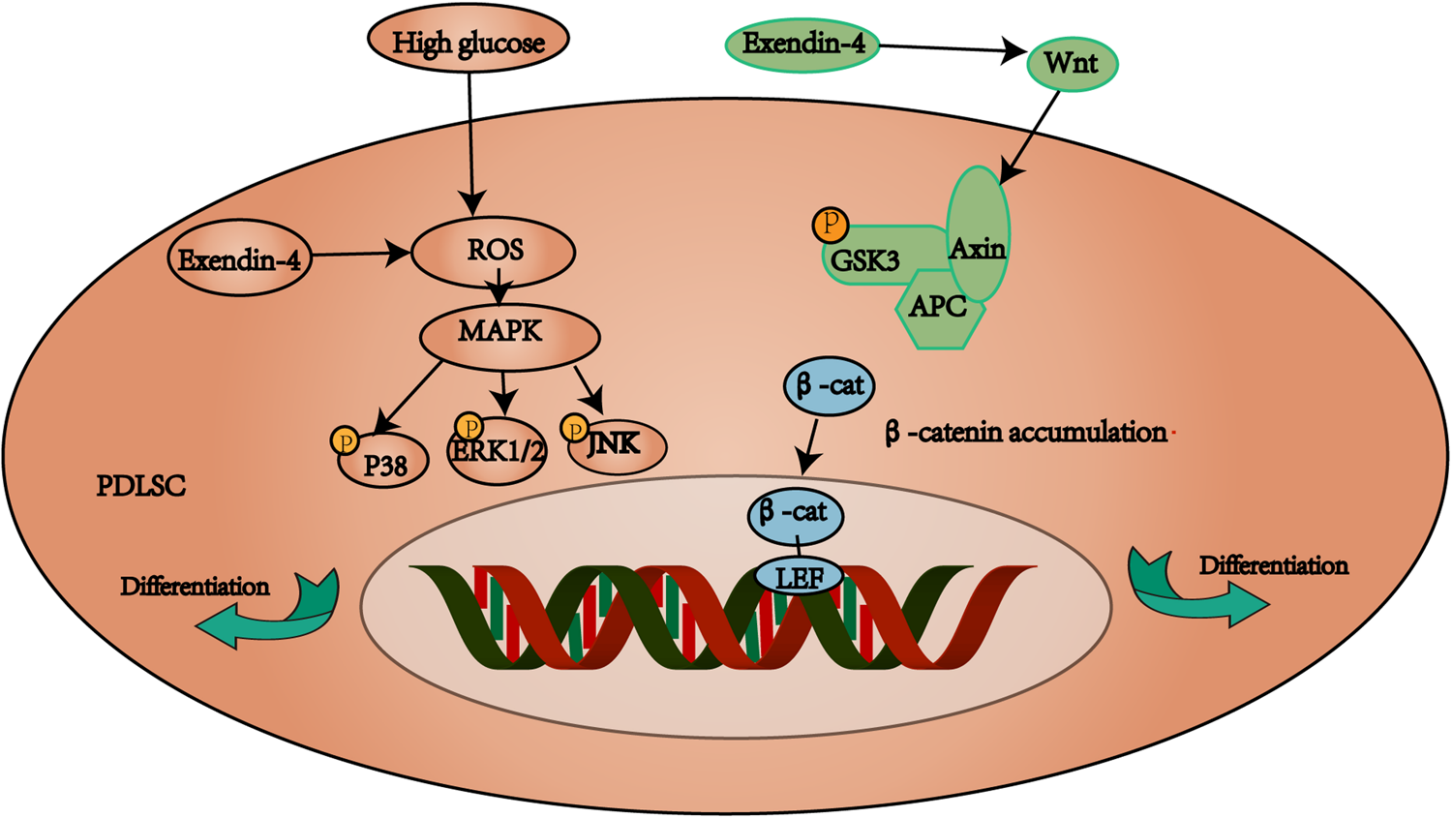
Hypothetical model of the signaling pathway through which Ex-4 alleviates osteogenesis in PDLSCs under a high glucose environment. High glucose increases the production of reactive oxygen species, whereas Ex-4 inhibits the production of ROS and activates the MAPK and WNT pathways, thus promoting the osteogenic differentiation of PDLSCs.

Our experiments demonstrated that the osteogenesis of PDLSCs was inhibited in a high glucose environment, but Ex-4 (10 nM) alleviated the inhibition of osteogenesis in PDLSCs by regulating the MAPK and WNT signaling pathways (Figure 6). Our findings may provide a new therapeutic target for patients with periodontitis and diabetes.

## Figures and Tables

**Figure A1:**
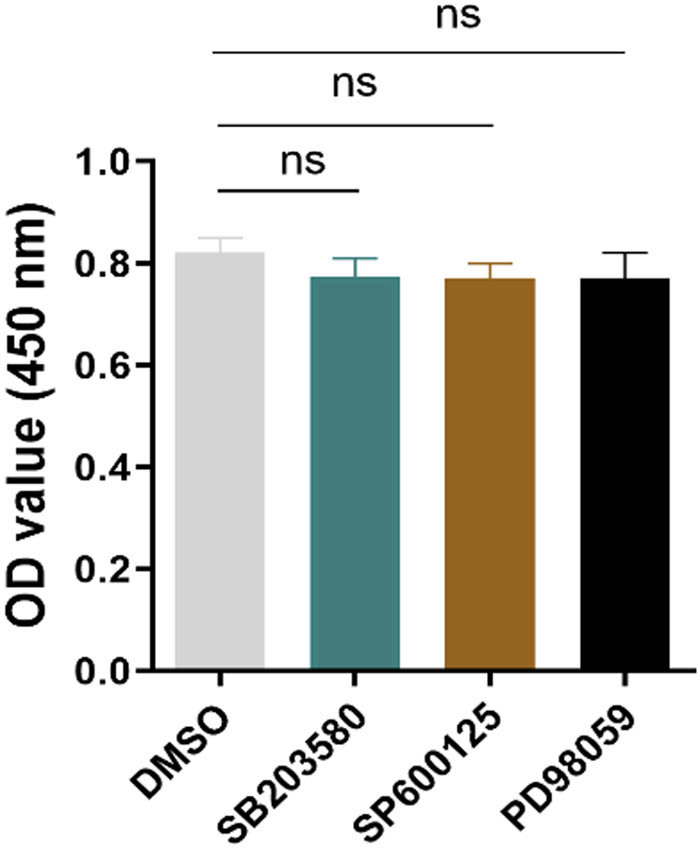
Effect of MAPK inhibitor concentration on PDLSCs cell viability.

**Figure A2:**
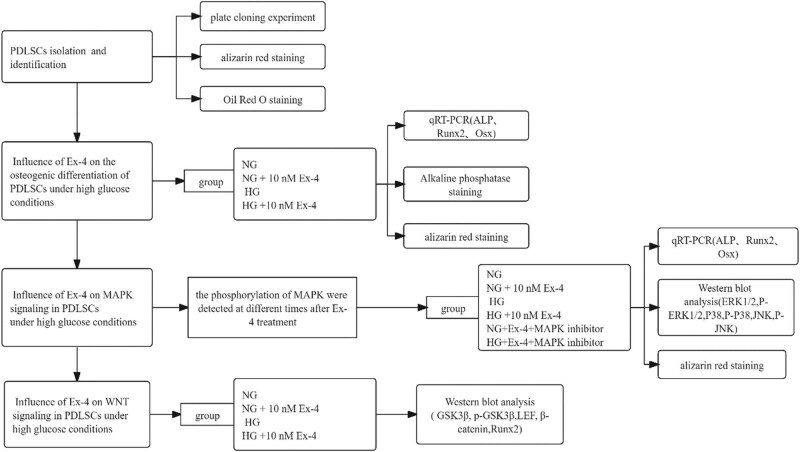
A workflow chart of the experiment.
